# Selection of Reference Genes for Gene Expression Studies Related to Intramuscular Fat Deposition in *Capra hircus* Skeletal Muscle

**DOI:** 10.1371/journal.pone.0121280

**Published:** 2015-03-20

**Authors:** Wuzheng Zhu, Yaqiu Lin, Honghai Liao, Yong Wang

**Affiliations:** College of Life Science and Technology, Southwest University for Nationalities, Chengdu 610041, Peoples’ Republic of China; University of Lleida, SPAIN

## Abstract

The identification of suitable reference genes is critical for obtaining reliable results from gene expression studies using quantitative real-time PCR (qPCR) because the expression of reference genes may vary considerably under different experimental conditions. In most cases, however, commonly used reference genes are employed in data normalization without proper validation, which may lead to incorrect data interpretation. Here, we aim to select a set of optimal reference genes for the accurate normalization of gene expression associated with intramuscular fat (IMF) deposition during development. In the present study, eight reference genes (*PPIB*, *HMBS*, *RPLP0*, *B2M*, *YWHAZ*, *18S*, *GAPDH* and *ACTB*) were evaluated by three different algorithms (geNorm, NormFinder and BestKeeper) in two types of muscle tissues (*longissimus dorsi* muscle and *biceps femoris* muscle) across different developmental stages. All three algorithms gave similar results. *PPIB* and *HMBS* were identified as the most stable reference genes, while the commonly used reference genes *18S* and *GAPDH* were the most variably expressed, with expression varying dramatically across different developmental stages. Furthermore, to reveal the crucial role of appropriate reference genes in obtaining a reliable result, analysis of *PPARG* expression was performed by normalization to the most and the least stable reference genes. The relative expression levels of *PPARG* normalized to the most stable reference genes greatly differed from those normalized to the least stable one. Therefore, evaluation of reference genes must be performed for a given experimental condition before the reference genes are used. *PPIB* and *HMBS* are the optimal reference genes for analysis of gene expression associated with IMF deposition in skeletal muscle during development.

## Introduction

Meat from goats is becoming more widely accepted around the world due to the increasing demand for sustainable foods and the low cholesterol content and high nutritive value of goat meat [1]. IMF content, the meat quality trait with the most economic importance [2], has a positive impact on meat characteristics such as tenderness, flavor and juiciness [3]. Thus, understanding the mechanism underlying IMF accumulation in skeletal muscle is of great importance to meat science. Gene expression analysis is a useful technique because it provides information on the regulation of IMF accumulation.

qPCR, with its high sensitivity, specificity and accuracy as well as ease of use [4], has been widely employed as the method of choice to characterize expression profiles of genes of interest and to verify results from microarray studies. Nevertheless, qPCR requires data normalization to correct for variability in the amount and quality of starting material, RNA stability, content, enzymatic efficiencies, and technique in the qPCR experimental process [5]. To address these problems, several strategies have been implemented for data normalization including quantifying RNA input or number of cells used. However, these methods are questionable because they either do not take into the consideration the imbalance in the abundance of mRNA and rRNA or ignore the efficiency of reverse transcriptase [6,7]. In addition, the amount of RNA maybe insufficient at times and accurate computation of cell number is often infeasible. Another proposed strategy is to use reference genes in data normalization; this strategy is currently considered a robust approach in most cases [8]. Reference genes are supposed to be constitutively expressed, and their expression should remain stable irrespective of experimental conditions such as developmental stage, experimental treatment, physiological state and tissue type [9,10]. However, there is an increasing number of reports demonstrating that the expression of most reference genes–including some commonly used reference genes such as *GAPDH*, *ACTB* and *18S*–is context dependent [5,11], as their expression may vary significantly depending on the experimental conditions being investigated [12]. No reference genes appear to be generally applicable for all circumstances. Accordingly, the validity of candidate reference genes has to be well established to circumvent problems in data normalization.

To date, several studies have attempted to identify suitable reference genes in ruminants. In a previous study on bovine intramuscular fat, *RPLP0* was validated as the most stable reference gene in bovine muscle across bovine breeds and ages [13]. In another study, Pérez et.al. [14] suggested that *SF3A1*, *EEF1A2* and *HMBS* were the most stable reference genes in bovine muscular tissue. Bonnet et.al. [15] demonstrated that *UXT*, *EIF3K*, *TBP*, *TOP2B* and *CLN3* were the best options for data normalization in bovine muscle, liver, mammary gland and adipose tissue. More recently, Najafpanah MJ et.al. [16] carried out an analysis of nine candidate reference genes in goat; *HSP-90* was almost always the most stable reference gene in *longissimus* muscle, liver and visceral and subcutaneous fat.

However, appropriate reference genes for use in the study of goat muscle, especially skeletal muscle during development, are lacking. Furthermore, only a few reports [17,18] on intramuscular fat deposition have appropriately evaluated reference genes in ruminants, and data on qPCR normalization are scarce in *Capra hircus*. Given the underlying issues associated with the use of non-validated reference genes for data normalization, in this study, we aimed to identify a set of reference genes that could serve as proper reference genes in *Capra hircus* skeletal muscle. Evaluation of eight reference genes was performed in skeletal muscle across four developmental stages. The geNorm [19], NormFinder [20] and BestKeeper [21] algorithms were applied to assess the stability of reference gene expression; qPCR procedures were performed following Minimum Information for Publication of Quantitative Real-Time PCR Experiments (MIQE) guidelines [22].

## Materials and Methods

### Animal and sample collection

All animal procedures were performed according to protocols approved by the Southwest University for Nationalities Animal Care and Use Committee in Sichuan, China.

Twenty-four male Jianyang big-eared goats, belonging to a meat goat breed developed from crossbreeding Nubian goats with Chinese local breeds, were selected for the experiment. All animals (average BW of 14.45±1.21 kg, mean age of 2 months) were castrated by a licensed veterinarian and then housed in four adjacent pens, with six goats per pen. All goats were raised under standard conditions, fed twice a day (08:30 and 17:00) and given free access to water. Goats were slaughtered at 3, 5, 7 and 12 months of age. At each time point, six goats were slaughtered. Slaughters were performed by exsanguination at a commercial abattoir owned by Sichuan AUSDA Husbandry Development Co., Ltd. *longissimus dorsi* muscle and *biceps femoris* musclewere dissected, rinsed with RNase-free water and frozen in liquid nitrogen until RNA isolation.

### RNA preparation and cDNA synthesis

Total RNA was extracted using Trizol (Invitrogen, Shanghai, China) according to the manufacturer’s protocol. RNA concentration was measured using a NanoDrop ND-1000 spectrophotometer (Agilent). Purity of the total RNA was determined by A260/280 and A260/230 ratios. RNA quality was assessed via agarose gel electrophoresis.

In our study, the A260/280 and A260/230 ratios ranged from 1.92 to 2.04 and from 2.01 to 2.12, respectively, which indicated the samples were of good quality. For each sample, 1μg of total RNA was reverse transcribed for cDNA synthesis using QuantiTect Reverse Transcription Kit (Qiagen) according to the manufacturer’s protocol. Prior to reverse transcription, genomic DNA was removed from the RNA with a specific gDNA Wipeout Buffer as described in the protocol.

### Selection of reference genes and primer design

Eight candidate reference genes belonging to different functional classes, most of which have been evaluated as reference genes in ruminants (goat [23], sheep [24], cattle [25]) and other livestock species (pig [26], chicken [27]) as well as in humans [28], were tested for stability. *HMBS*, *PPIB* and *RPLP0* were chosen because they had been previously reported to be the best reference gene in bovine muscle tissues [14,17,29]. *B2M* and *YWHAZ* were selected because they had been identified as suitable reference genes in porcine *longissimus thoracis et lumborum* (LTL) muscle and frozen whole blood of goats, respectively [23,30]. Additionally, *GAPDH*, *18S* and *ACTB* were included because these genes have been utilized as reference genes in numerous studies [31,32,33].

Prior to primer design, a pretest study was conducted with primers identified by literature review for several of the reference genes. However, the performance of some of the primers was not as good as expected, and the optimal annealing temperatures of the primers are not identical. Thus, assays using these primers may work poorly and, furthermore, cannot run simultaneously on the same plate, making the results vulnerable to inter-assay variation. To reduce the inter-assay variation as much as possible for improved qPCR performance and to provide a set of species-specific primers for reference genes in goat, primer pairs were designed by primer5.0 software on the basis of caprine sequence information available in GenBank. Primer details are listed in Tables 1 and 2. Prior to RT-qPCR, conventional PCR and agarose gel electrophoresis were performed to test the gene-specific primers and verify the amplified products. All PCR products were sequenced and then aligned against the goat genome with the BLAST program to verify their identity. The specificity of the primers was evaluated by melting curve analysis. Only primers producing a single band of the expected size whose sequence matched published sequences exactly and exhibiting a unique peak during the dissociation step of the melting curve analysis were used for RT-qPCR.

**Table 1:** Primers and relative information of reference and target genes.

Gene Symbol	Accession number	Sense primer sequence 5′ to 3′	Anti-sense primer sequence 5′ to 3′	Amplication size (bp)	T_m_ (°C)
*PPIB*	XM_005685667	ACACCAACGGCTCCCAGT	AGGCTTGTCCCGACCATC	143	60
*RPLP0*	XM_005709526	TTCTCCTTCGGGCTGGTCA	TCCAGGAAGCGGGAATGC	104	60
*HMBS*	AB232537	GCGGGAGAGCCCCTATGA	AGCTGCGGCCAGGATGAT	230	60
*B2M*	DQ386890	TGTCCCACGCTGAGTTCACT	TGAGGCATCGTCAGACCTTGA	137	60
*GAPDH*	AJ431207	GCAAGTTCCACGGCACAG	TCAGCACCAGCATCACCC	118	60
*18S*	DQ149973	TAATCCCGCCGAACCCCATT	GGTGTGTACAAAGGGCAGG	125	60
*ACTB*	JX046106	CTTCCAGCCGTCCTTCCT	TGTTGGCATACAGGTCCTTTC	105	60
*YWHAZ*	NC_022306	AGGAGCCCGTAGGTCATCTTG	CGAGCCATCTGCTGTTTTTTC	84	60
*PPARG*	JQ266369	ACGGGAAAGACGACAGACAAA	CTGACACCCCTGGAAGATGC	147	60

**Table 2:** Description of reference and target genes analyzed in this study.

Gene Symbol	Gene name	Function	Efficiency	R^2^	slope
*PPIB*	Peptidylprolyl isomerase B	Protein folding catalyst	104.0%	0.999	−3.228
*RPLP0*	Acetic ribosomal protein large P0	Protein synthesis	98.9%	1.000	−3.348
*HMBS*	Hydroxymethyl-bilane synthase	Heme biosynthesis	96.2%	0.999	−3.415
*B2M*	Beta-2-Microglobulin	Cell surface molecule component	96.0%	0.999	−3.421
*GAPDH*	Glyceraldehyde-3-phosphate dehydrogenase	Glycolytic enzyme	100.0%	1.000	−3.321
*18S*	18S Ribosomal RNA	Ribosomal RNA	98.3%	0.999	−3.364
*ACTB*	Beta-actin	Cytoskeletal structural protein	100.1%	1.000	−3.320
*YWHAZ*	Tyrosine 3-monooxygenase	Signal transduction	98.2%	0.999	−3.365
*PPARG*	peroxisome proliferator-activated receptor gamma	Lipid metabolism	97.6%	0.998	−3.382

### Quantitative Real Time RT-PCR (qPCR)

Real-time PCR reactions were performed in 96-well plates with a Bio-Rad CFX96TM Real-time PCR Detection System. Reactions were carried out in a final volume of 25 μl containing 12.5μl 2× QuantiFast SYBR Green PCR Master Mix (Qiagen), 500nM of each primer, 1μl cDNA and 9.5μl DNase/RNase-free water. The cycling program was 95°C for 5 min to activate the polymerase followed by 40 cycles of denaturation at 95°C for 10s, annealing at 60°C for 20s and extension at 72°C for 20s. Melting curve analysis was conducted by heating samples from 65°C to 95°C with continuous fluorescent acquisition. All reactions were performed in triplicate for each cDNA sample. In addition, no template controls were tested for each primer pair. Standard curves were established using a 10-fold dilution series of purified PCR fragments as templates.

### Data analysis

Three classic software programs, geNorm, NormFinder and BestKeeper, each with a unique advantage, were used to evaluate the reference genes.

geNorm defines the standard deviation (SD) of the expression ratio of two reference genes as a pairwise variation under the assumption that the genes tested are not co-regulated and that the expression ratio is identical across all samples. The gene-stability measure (*M*) is then calculated as the average pairwise variation of a specific gene compared with all of the other reference genes. Genes with the highest M values have the least stable expression. Eventually, all candidate reference genes are ranked by stepwise elimination of the genes with the highest *M* value. The outstanding advantage of geNorm is that it can identify the number of genes required for dependable normalization.

NormFinder is a model-based approach in which two types of variation are utilized to determine systematic error. This method takes into account both intragroup and intergroup variation; the latter is commonly overlooked in pairwise methods.

In contrast to geNorm and NormFinder, BestKeeper uses raw data instead of relative quantities. The estimate of reference gene stability is calculated based on the SD values of each gene. Then, the BestKeeper index is calculated from the geometric mean of the candidates C_T_ values for each specific sample. The most stable reference genes are the ones with the lowest SD values and highest coefficients of correlation with the BestKeeper index.

To evaluate the efficacy of the selection of reference genes for normalization, the relative change in the expression of target gene (*PPARG*) mRNA in skeletal muscle was calculated across different developmental stages using the 2^−ΔΔCT^ method [34], in which ΔΔCT = [(C_T *PPARG*_−C_T reference gene_)_month x_−(C_T *PPARG*_−C_T reference gene_)_month 12_]. Normalization was carried out with the least stable reference genes and the geometric mean of the most stable reference genes.

All relative quantification data were analyzed by one-way ANOVA with developmental stage as a factor using the PROC ANOVA procedure of the SAS software package, version 9.0. Duncan’s multiple-range test was performed for multiple comparisons when one-way ANOVA results were significant. Differences of *P<0*.*05* were considered significant.

## Results

### Primer validation

For all primer pairs, the amplification efficiency ranged between 96% and 104% and the coefficient of determination (R^2^) varied from 0.998 to 1 (Table 2). Single peaks were observed in the analysis of melting curves, and agarose gel electrophoresis revealed a unique band at the expected size in the gel. In addition, the sequences of the amplified DNA fragments matched the reference and target gene sequences available in GenBank. Specific details of primer pair validation are presented in Supporting Information.

### Expression profiling of candidate reference genes

**Fig 1:**
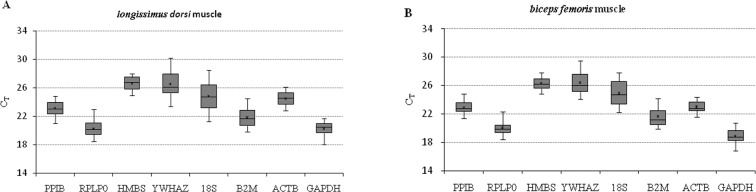
The distribution of reference gene expression levels in *longissimus dorsi* muscle (A) and *biceps femoris* muscle (B). Means and medians are indicated by asterisks and lines, respectively. The boxes encompass the 25th to the 75th percentiles. Whisker caps denote the maximum and minimum values.

The expression of eight candidate reference genes was assayed in samples of *longissimus dorsi*muscle and *biceps femoris* muscle. The distribution of C_T_ values is displayed in Fig. 1. Mean C_T_ values ranged from 20.19 to 26.56 in *longissimus dorsi* muscle and 18.48 to 26.41 in *biceps femoris*muscle and provide an overview of the variation in the expression of the candidate reference genes. Though average C_T_ values varied, the overall distribution of the C_T_ values was similar in *longissimus dorsi* muscle and *biceps femoris* muscle. This may be because both tissues are types of skeletal muscle.

### GeNorm Analysis

**Fig 2:**
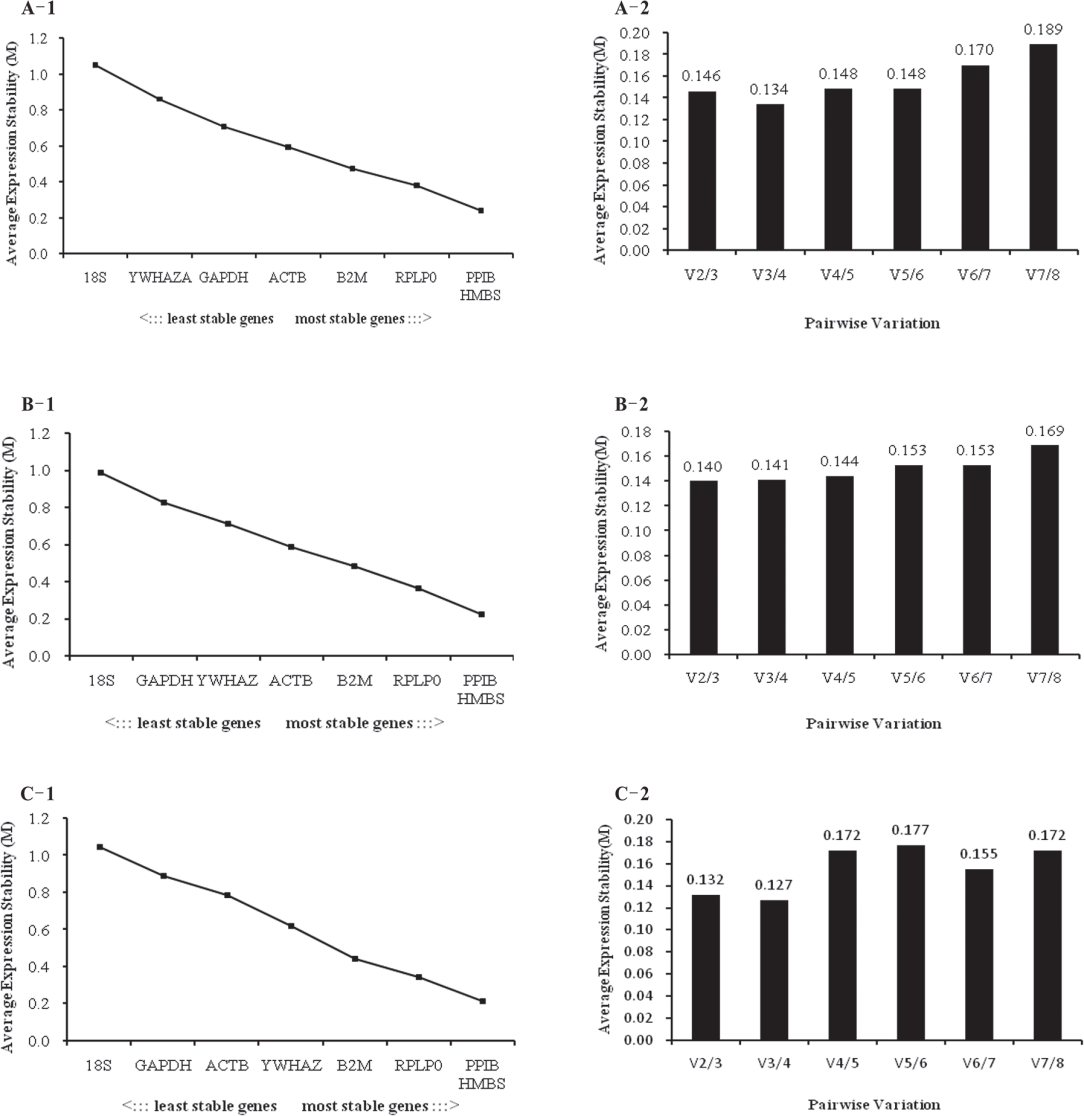
Gene expression stabilities and rankings of reference genes as calculated by geNorm. Rank-order of gene expression stability is shown for *longissimus dorsi* muscle (A-1), *biceps femoris* muscle (B-1) and the combined group (C-1) according to the average expression stability values (*M*) for the reference genes from the least stable (left) to the most stable (right). Pairwise variation analysis (*V*) to determine the optimal number of reference genes for data normalization in *longissimus dorsi* muscle (A-2), *biceps femoris* muscle (B-2) and the combined group (C-2).

The ranking of candidate genes was determined by geNorm software on the basis of their stability. In geNorm analyses, genes with *M* values below the cut-off value of 1.5 are considered to be stable, while those with *M* values above the threshold should not be used for data normalization. Fig. 2 shows the *M* values of all candidate genes during development for *longissimus dor*si muscle, *biceps femoris* muscle and the combined group. All candidate genes evaluated in our study exhibited *M* values below 1.1, indicating acceptable expression stability. With the lowest M values, *PPIB* and *HMBS* had the most stable gene expression during the development of both *longissimus dorsi* muscle and *biceps femoris* muscle, whereas *18S* exhibited significant variation and was the least stable gene. *PPIB* and *HMBS* were also the most stable genes when all samples were analyzed together. Similarly, *18S* remained the least stable gene with the highest score in the combined group. In addition, the commonly used reference genes *GAPDH and YWHAZ* were not stably expressed in any sample. *GAPDH* was particularly variable, ranking just below *18S* in *biceps femoris* muscle and the combined group. Hence, small differences existed in the geNorm rank of reference genes in the different muscle types.

The optimal number of reference genes needed for accurate normalization was determined by geNorm on the basis of the average pairwise variation (*Vn/n+1*) value. Our cut-off value was 0.15. Below this value, inclusion of an additional reference gene offers little further improvement to the data normalization. As depicted in Fig. 2, the value of V2/3 fell below 0.15 for all groups, implying that two reference genes—*PPIB* and *HMBS*—would be sufficient for accurate normalization across all samples.

### NormFinder Analysis

NormFinder can identify the optimal reference gene with the most stable expression. As illustrated in Table 3, *PPIB*, having the lowest stability value in all groups, was again identified as the most stable gene, while the second-most stable gene was *B2M* in *longissimus dorsi* muscle and *HMBS* in *biceps femoris* muscle and the combined group. *18S* exhibited the worst stability of the eight tested reference genes in all sample sets. Although *ACTB* and *RPLP0* exhibited moderate variability, the commonly used reference genes *GAPDH* and *YWHAZ* tended to rank near the bottom of the candidate genes, which is in agreement with the results generated by geNorm analysis.

**Table 3:** Ranking and stability values of reference genes calculated by NormFinder.

	*Longissimus dorsi* muscle		*Biceps femoris* muscle		Combined group	
Rank order	Gene name	Stability Value	Gene name	Stability Value	Gene name	Stability Value
1	*PPIB*	0.083	*PPIB*	0.077	*PPIB*	0.074
2	*B2M*	0.213	*HMBS*	0.176	*HMBS*	0.208
3	*HMBS*	0.256	*ACTIN*	0.408	*B2M*	0.317
4	*ACTIN*	0.353	*B2M*	0.422	*RPLP0*	0.469
5	*RPLP0*	0.538	*RPLP0*	0.468	*ACTIN*	0.568
6	*GAPDH*	0.644	*YWHAZ*	0.627	*YWHAZ*	0.663
7	*YWHAZ*	0.766	*GAPDH*	0.656	*GAPDH*	0.708
8	*18S*	1.035	*18S*	0.919	*18S*	0.937

### BestKeeper Analysis

In BestKeeper, repeated pairwise correlation analysis was performed to determine the optimal reference genes. The premise of BestKeeper is that the SD values of any studied genes should not be higher than 1. The more stable genes have Pearson coefficients of correlation (R) close to1. *YWHAZ*, *18S* and *B2M* exhibited substantial variation in all sample groups with SD values exceeding 1 (S3 Table). Thus, these genes were excluded and the analysis repeated. *GAPDH* was also excluded from the combined group because its SD value was 1. Table 4 displays the results for the remaining genes. *PPIB* and *HMBS* ranked as the two most stable reference genes in all sample sets with R-values ranging from 0.957 to 0.981. In addition, BestKeeper allows for the analysis of reference genes and target gene at same time. *PPARG* exhibited considerable variation in both *longissimus dorsi* muscle and *biceps femoris* muscle, with SD values of 1.37 and 1.12, respectively (S4 Table), demonstrating that *PPARG* is expressed variably during skeletal muscle development.

**Table 4:** Reference genes stability calculated by BestKeeper based on C_T_.

*Longissimus dorsi* muscle			*Biceps femoris* muscle			Combined group		
Gene	Coeff. of corr. [R]	Std dev [± C_T_]	Gene	Coeff. of corr. [R]	Std dev [± C_T_]	Gene	Coeff. of corr. [R]	Std dev [±C_T_]
*PPIB*	0.968	0.80	*PPIB*	0.981	0.76	*HMBS*	0.972	0.60
*HMBS*	0.968	0.60	*HMBS*	0.957	0.58	*PPIB*	0.969	0.78
*ACTIN*	0.889	0.76	*ACTIN*	0.924	0.47	*RPLP0*	0.873	0.66
*GAPDH*	0.781	0.77	*RPLP0*	0.756	0.65	*ACTIN*	0.749	0.84
*RPLP0*	0.775	0.68	*GAPDH*	0.658	0.61			

Abbreviations: Coeff. of corr. [R]: Pearson coefficient of correlation; Std dev [± C_T_]: the standard deviation of the C_T_.

### Reference gene validation

To determine whether there is a significant difference between target gene expression normalized to the most stable reference genes and that normalized to the least stable reference genes, we analyzed the expression of *PPARG* at different developmental stages normalized to *GAPDH*, *18S* and the geometric mean of *PPIB* and *HMBS*.

*PPARG* is an important regulator of many genes involved in adipogenesis [35] and is mainly expressed in intramuscular connective tissues in goat [36]. In vitro, up-regulation of *PPARG* allows for differentiation of pre-adipocytes into mature adipocytes in bovine adipose tissue, which is essential for promoting adipogenesis [37]. Additionally, some of the genes required for the terminal differentiation of adipocytes are under the control of *PPARG* [37]. *PPARG* is also involved in sustaining the adipocyte differentiation program and is an activator of fatty acid synthesis and storage [38], which is crucial to intramuscular fat deposition. Because of its critical role in intramuscular fat development, *PPARG* was chosen as the target gene for our study.

**Fig 3:**
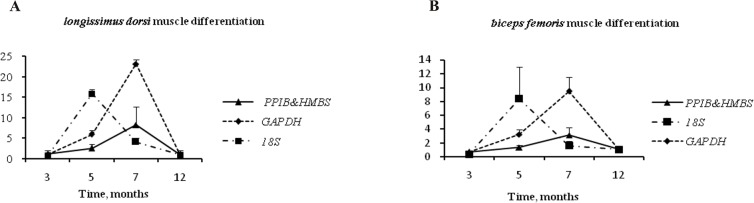
Effect of normalization on PPARG gene expression in skeletal muscles. The expression of *PPARG* was normalized to the geometric mean of *PPIB*&*HMBS*, *18S* or *GAPDH* expression and is shown relative to expression at 12 months for *longissimus dorsi* muscle (A) and *biceps femoris* muscle (B). Error bars indicate SD.

Our results suggest that the expression of *PPARG* varies during the development of skeletal muscle, which is consistent with the findings of our BestKeeper analysis. In both *longissimus dorsi* muscle and *biceps femoris* muscle, the expression pattern of *PPARG* across different developmental stages was similar when normalized to either *PPIB&HMBS* or *GAPDH*, whereas the expression pattern of *PPARG* was distinguishably different when normalized to *18S* (Fig. 3). In *longissimus dorsi* muscle, compared with normalization against *PPIB&HMBS*, the most stably expressed genes, normalization against *GAPDH* led to a dramatic up-regulation of *PPARG* expression, with up to 2.8-fold changes observed at the peak of relative expression at 7 months. When *PPARG* expression was normalized to *18S*, the peak of the expression occurred at 5 months, with up to 6.2-fold changes in expression compared with normalization against *PPIB&HMBS*. Similar results were detected in *biceps femoris* muscle.

## Discussion

Appropriate reference genes are indispensible for accurate data normalization and thus reliable results in studies of gene expression. However, numerous studies choose reference genes without proper validation, picking genes that have been used for qPCR analyses in other investigations or arbitrarily selecting commonly used reference genes (*eg*: *GAPDH*, *ACTB* and *18S*). Unfortunately, the use of such empirical reference genes is problematic, as increased variability may occur under certain experimental conditions [39,40]. Moreover, many published studies have shown that a number of commonly used reference genes, which are subjectively thought to be invariable irrespective of experimental conditions and have been used in published IMF research [41,42,43], are regulated and can vary considerably [44,45,46]. Thus, there is no doubt that reference genes appropriate for a certain context may differ from those suitable for other circumstances and that universal reference genes cannot exist. Thus, proper validation of reference genes should be performed prior to data normalization.

In this study, eight candidate reference genes have been evaluated for stability across a number of developmental stages in both *longissimus dorsi* muscle and *biceps femoris* muscle. Several algorithms with different working rationales, such as geNorm, NormFinder and BestKeeper, have been developed to facilitate selection of appropriate reference genes. geNorm concentrates on pairwise comparisons of genes with the highest degree of similarity in the expression profile, considering only intergroup variation. Thus, the geNorm algorithm may not work well when co-regulated genes are included. Unlike geNorm and Bestkeeper, which are based on pairwise comparisons, NormFinder takes both intragroup and intergroup variations into account in the stability value it calculates for each candidate gene, which causes it to be less sensitive to genes with correlated expression. A direct evaluation of expression variation is performed for every candidate gene in each sample with NormFinder, testing each gene in a given context [20]. NormFinder, unlike geNorm, does not determine the optimal number of reference genes. BestKeeper enables the user to analyze reference and target genes simultaneously. For increased reliability, more than one type of algorithm should be used to evaluate reference genes because algorithms have different strengths and weakness.

To prevent possible bias, three complementary algorithms (geNorm, NormFinder and BestKeeper) were used in our study. Despite the differences between the algorithms, the results obtained from all three algorithms were consistent. *PPIB* and *HMBS* were found to be the most stable genes during the development of *longissimus dorsi* muscle and *biceps femoris* muscle, regardless of whether the samples were analyzed separately or together. *PPIB* was previously validated as a reference gene for studies on IMF deposition in beef [17]. In addition, *PPIA*, which belongs to the same group of cyclophilin proteins as *PPIB* [47], has been shown to be the best reference gene for *longissimus dorsi* muscle development in pig [48]. Thus, identical results were observed in our research and in previous studies, demonstrating that *PPIB* is an appropriate normalization factor in skeletal muscle. *HMBS* outperformed all of the candidates aside from *PPIB* and is regarded as an optimal reference gene in bovine skeletal muscle [14] and multiple goat tissues [49]. The reference genes *RPLP0* and *B2M*, which are the most stable genes under some conditions [15,23,50], displayed only moderate stability in our study, suggesting that the best reference genes for one environment may be unsuitable for another. Our geNorm results indicate that the two reference genes with the most stable expression in all groups (*PPIB* and *HMBS*) are sufficient for data normalization. However, if more than two reference genes are used, as Vandesompele recommends [19], *RPLP0* may be a good choice. *RPLP0* ranked as the third-most stable gene in all groups in our geNorm analysis and was identified as the optimal reference gene for research involving IMF development in cattle [29] and milk somatic cells in goat [23]. *18S* and *GAPDH* were the least stable genes tested, ranking in the bottom half of the list in all analyses. As such, neither are appropriate reference genes for skeletal muscle studies.

To determine the impact normalization to different reference genes has on the expression pattern of target genes, the relative expression of *PPARG* was evaluated by normalizing it to several reference genes. The expression of *PPARG* in different muscle types, normalized against *GAPDH* and *PPIB&HMBS*, followed a similar pattern during development. However, an obvious overestimation of *PPARG* expression changes occurred when *PPARG* was normalized to *GAPDH* rather than *PPIB&HMBS*. When *18S* was used in the normalization, significant fold changes in expression were detected at 5 and 7 months. Therefore, our comparison revealed substantial differences in the relative expression levels of *PPARG* when different genes were used for normalization, demonstrating the need for accurate validation of reference genes. Particular attention should be paid to commonly used reference genes, as inappropriate usage could lead to misinterpretation of results. In addition, when *PPIB* and *HMBS* were independently utilized for normalization, the relative abundance of *PPARG* was not altered markedly (data not shown). This maybe because there is little difference in their stability; both genes exhibited stable expression in all analyses by the three algorithms.

**Fig 4:**
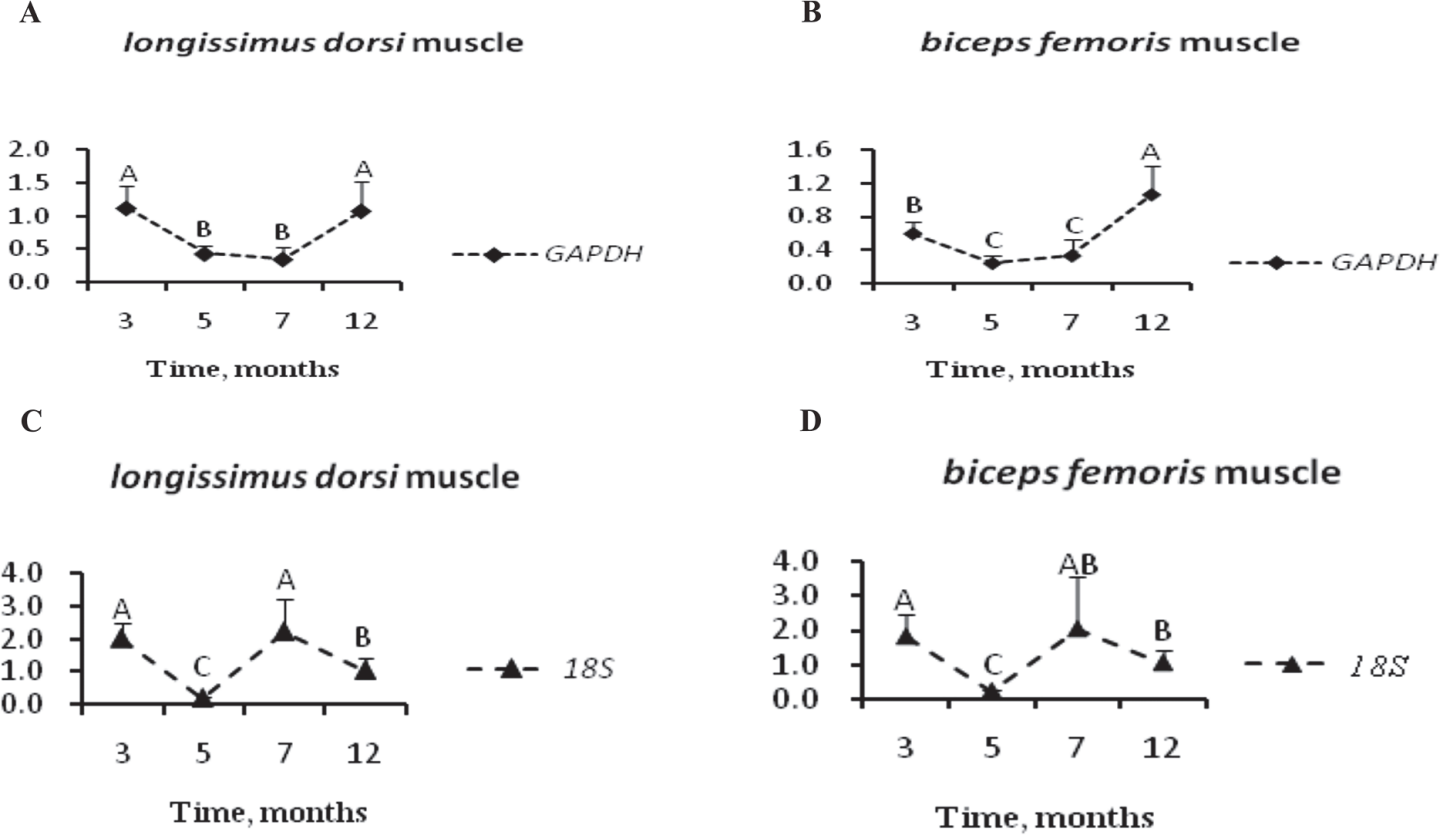
Expression profiling of *GAPDH* and *18S* in skeletal muscles at different developmental stages. Gene expression data for both *longissimus dorsi*(A、C) and *biceps femoris* muscle (B、D)were normalized to the geometric mean of *PPIB&HMBS* and are relative to expression at 12 months following the 2^−ΔΔCT^ method [34]. Error bars depict SD. Superscript capital letters indicate significant differences (*P<0*.*05*).

Given the differences observed in our comparison, we conclude that normalization against a single “commonly used reference gene” may lead to serious discrepancies in data interpretation. *GAPDH* was found to be unsuitable for normalization in skeletal muscle development in pig [48]; similar results were reported in goat [16] and cattle [51]. In addition, *GAPDH* was observed to be up-regulated and down-regulated under various conditions [7,9,52,53]. Likewise, a remarkable change in the expression of *18S* occurred during osteoblast differentiation, with a continual increase in expression observed over time [6]. Given the afore mentioned influence of the environment on *GAPDH* expression and the distinct impact normalization to *GAPDH* had on the relative expression level of *PPARG*, we hypothesized that there are great fluctuations in the expression of *GAPDH* and *18S* over the course of development. To test this hypothesis, relative expression of *18S* and *GAPDH* was plotted by normalizing to the geometric average of *PPIB&HMBS*. As illustrated in Fig. 4, in *longissimus dorsi* muscle we observed a pronounced decrease in *GAPDH* expression between 3 and 7 months followed by a sharp increase in expression. The expression pattern was opposite to that of *PPARG*. The dramatic regulation of *GAPDH* expression over the course of skeletal muscle development may account for the overestimation of the changes in *PPARG* expression when *GAPDH* was used for normalization. An even greater modulation of expression was seen over time when expression was normalized to *18S* because the relative expression level of *18S* was significantly different at all time points except 3 and 7 months. Similar results were observed in *biceps femoris* muscle. Thus, these data support our hypothesis that *GAPDH* and *18S* expression varies significantly during skeletal muscle development.

## Conclusions

In our study, three different algorithms were used to analyze a panel of reference genes. All three algorithms gave consistent results. *PPIB* and *HMBS* proved to be the optimal reference genes in both *longissimus dorsi* muscle and *biceps femoris* muscle during development. *GAPDH* and *18S* were the least stable genes, suggesting they are not suitable for expression data normalization in studies of goat muscle. Importantly, proper validation of reference genes is essential for reliable interpretation of data and should be performed prior to data normalization.

## Supporting Information

S1 Fig Melting curves and standard curves of eight reference genes and a target gene. (DOCX)

S2 Fig Agarose gel electrophoresis identification of gene-specific primers of reference genes for qPCR. (DOCX)

S1 Table Sequencing results of PCR products from the amplification of primers of reference and target genes designed for this experiment. (DOCX)

S2 Table Sequencing results of genes using BLASTN from NCBI against nucleotide collection (nr / nt). (DOCX)

S3 Table Reference genes stability calculated by BestKeeper based on C_T_. (DOCX)

S4 Table Descriptive statistics of target gene (*PPARG*) analyzed by BestKeeper in *Longissimus dorsi muscle* and Biceps femoris muscle, respectively. (DOCX)
