# Supplementary material for: Selection of Reference Genes for Gene Expression Studies Related to Intramuscular Fat Deposition in Capra hircus Skeletal Muscle
Source: PLoS One. 2015 Mar 20;10(3):e0121280. doi: 10.1371/journal.pone.0121280 (PMC4368700; doi:10.1371/journal.pone.0121280)
Supplement: S1 Fig — (DOCX) [file pone.0121280.s001.docx]

**Figure S1. Melting curves and standard curves of eight reference genes and a target gene.**


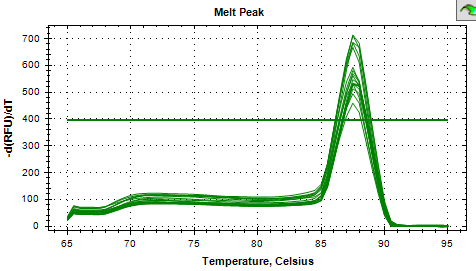

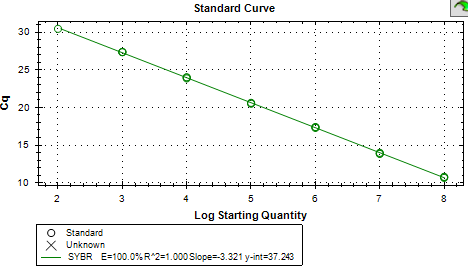


Figure A. Melting curve & standard curve of *GAPDH*


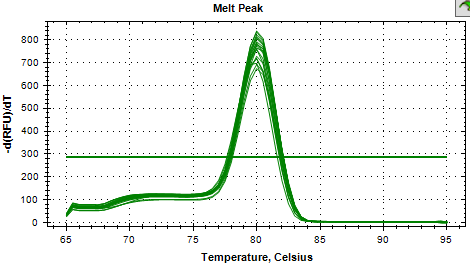

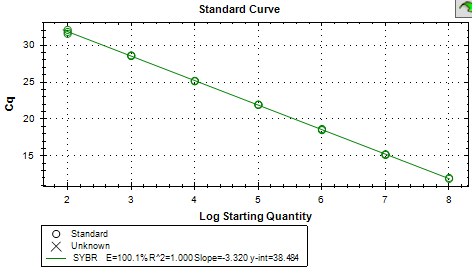


Figure B. Melting curve & standard curve of *ACTB*


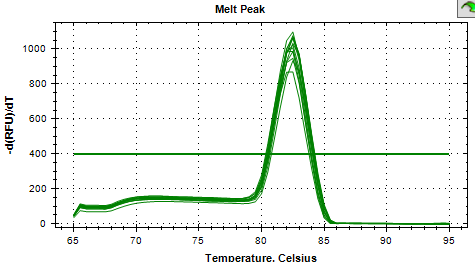

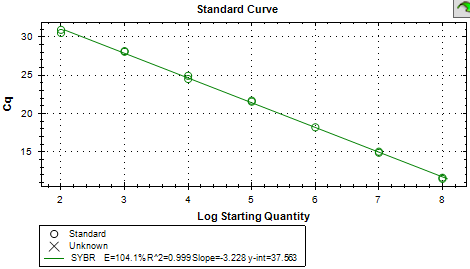


Figure C. Melting curve & standard curve of *PPIB*


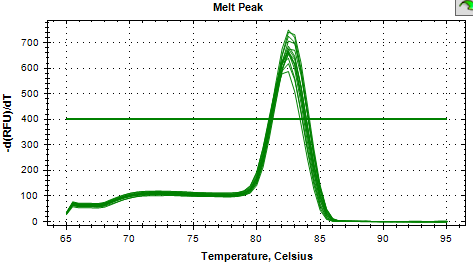

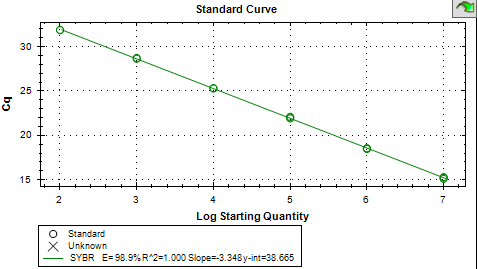


Figure D. Melting curve & standard curve of *RPLP0*


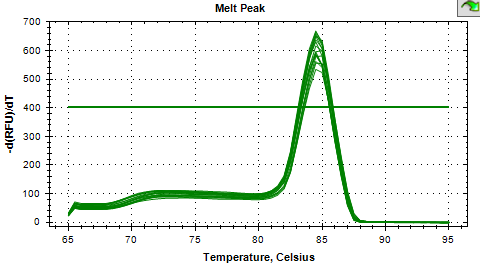

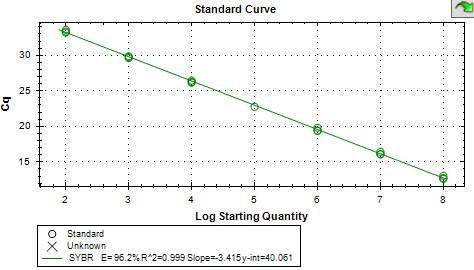


Figure E. Melting curve & standard curve of *HMBS*


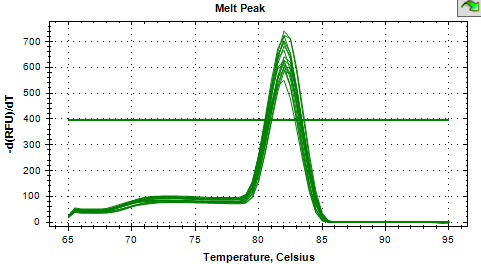

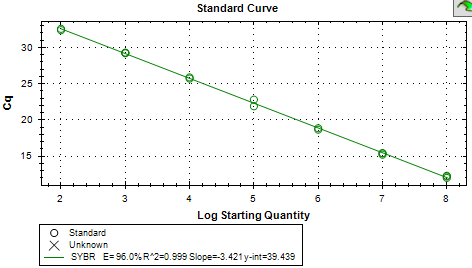


Figure F. Melting curve & standard curve of *B2M*


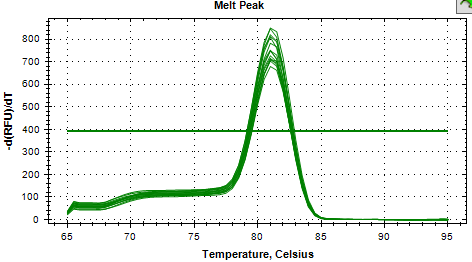

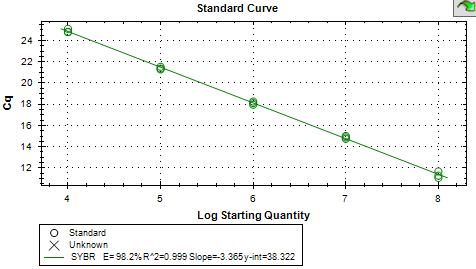


Figure G. Melting curve & standard curve of *YWHAZ*


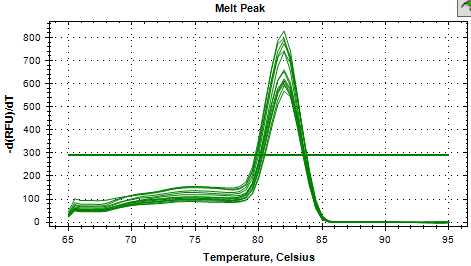

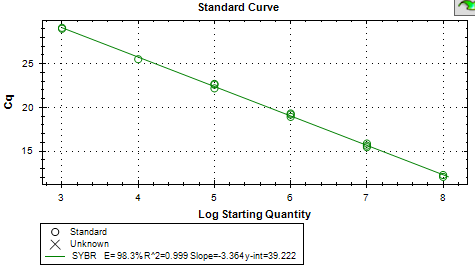


Figure H. Melting curve & standard curve of *18S*


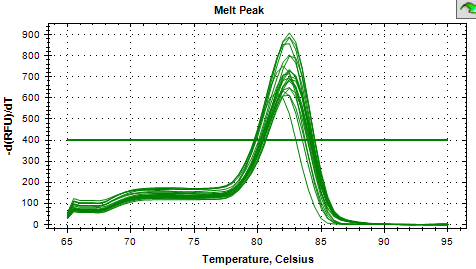

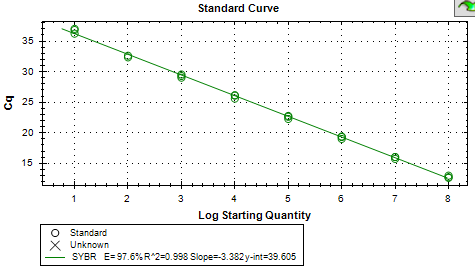


Figure J. Melting curve & standard curve of *PPARG*
