# Supplementary material for: Selection of Reference Genes for Gene Expression Studies Related to Intramuscular Fat Deposition in Capra hircus Skeletal Muscle
Source: PLoS One. 2015 Mar 20;10(3):e0121280. doi: 10.1371/journal.pone.0121280 (PMC4368700; doi:10.1371/journal.pone.0121280)
Supplement: S2 Fig — (DOCX) [file pone.0121280.s002.docx]

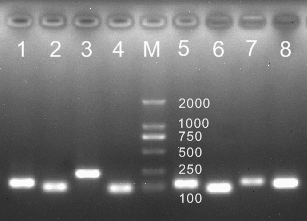


**Figure S2.** **Agarose gel electrophoresis identification of gene-specific primers of reference genes for qPCR.** Agarose gel (2%) electrophoresis showing amplification of specific band at the expected size for each reference gene. 1: *PPIB*, 2:*RPLP0*, 3: *HMBS*, 4: *YWHAZ*, M: D_2000_ DNA Marker (100-2000bp), 5: *18S*, 6: *ACTB*, 7: *B2M*, 8: *GAPDH*.
