# Supplementary material for: Selection of Reference Genes for Gene Expression Studies Related to Intramuscular Fat Deposition in Capra hircus Skeletal Muscle
Source: PLoS One. 2015 Mar 20;10(3):e0121280. doi: 10.1371/journal.pone.0121280 (PMC4368700; doi:10.1371/journal.pone.0121280)
Supplement: S1 Table — (DOCX) [file pone.0121280.s003.docx]

**Table S1. Sequencing results of PCR products from the amplification of primers of reference and target genes designed for this experiment.**

| **Gene** | **Sequence** |
| --- | --- |
| *PPIB* | ACACCAACGGCTCCCAGTTCTTCATCACGACAGTCAAGACTGCTTGGTTAGACGGCAAGCATGTAGTGTTTGGCAAAGTTCTAGAGGGCATGGATGTAGTACGGAAGGTAGAGAGCACCAAGACAGATGGTCGGGACAAGCCT |
| *RPLP0* | TTCTCCTTCGGGCTGGTCATCCAGCAGGTGTTTGACAATGGCAGCATCTACAACCCCGAAGTGCTTGACATCACCGAGGAAACTCTGCATTCCCGCTTCCTGGA |
| *HMBS* | GCGGGAGAGCCCCTATGATGCTGTTGTCTTTCACCCAAAATTTGTTGGGAAGACTCTAGAAACCTTGCCAGAGAAGAGTGTGGTAGGAACTAGCTCCCTGCGGAGAGCAGCCCAGCTGCAGAGAAAGTTCCCACACCTGGAGTTCAGGAATATTCGGGGAAACCTCAACACACGGCTGCGTAAGCTGGATGAGCTGCAGGAGTTCAGTGCCATCATCCTGGCCGCAGCT |
| *B2M* | TGTCCCACGCTGAGTTCACTCCCAACAGCAAGGATCAGTACAGCTGCCGAGTGAATCACGTTACTTTAACACAACCCAAGATAGTTAAGTGGGATCGAGACCTCTAAGCAGCACCATCAAGGTCTGACGATGCCTCA |
| *GAPDH* | GCAAGTTCCACGGCACAGTCAAGGCAGAGAACGGGAAGCTCGTCATCAATGGAAAGGCCATCACCATCTTCCAGGAGCGAGATCCTGCCAACATCAAGTGGGGTGATGCTGGTGCTGA |
| *18S* | TAATCCCGCCGAACCCCATTCGTGATGGGGATCGGTGGATGCATTATTCCCCATGAACGAGGAATTCCCAGTAAGTGCGGGTCATAAGCTTGCGTTGATTAAGTCCCTGCCCTTTGTACACACC |
| *ACTB* | CTTCCAGCCGTCCTTCCTGTATGGAGTCCTGTGGCATCCACGAGACCACCTTCAATTCCATCATGAAGTGTGACGTGGACATCCGAAAGGACCTGTATGCCAACA |
| *YWHAZ* | AGGAGCCCGTAGGTCATACTGGAGGGTCATCTCCAGTATTGAGCAAAAGACGGAAGGTGCTGAGAAAAAACAGCAGATGGCTCG |
| *PPARG* | ACGGGAAAGACGACAGACAAATCACCGTTTGTTATCTATGACATGAACTCCTTAATGATGGGAGAAGATAAAATCAAGTTCAAGCACATCAGCCCCCTGCAGGAGCCCAGCAAAGAGGTGGCCATCCGCATCTTCCAGGGGTGTCAG |
