# Supplementary material for: Selection of Reference Genes for Gene Expression Studies Related to Intramuscular Fat Deposition in Capra hircus Skeletal Muscle
Source: PLoS One. 2015 Mar 20;10(3):e0121280. doi: 10.1371/journal.pone.0121280 (PMC4368700; doi:10.1371/journal.pone.0121280)
Supplement: S2 Table — (DOCX) [file pone.0121280.s004.docx]

**Table S2. Sequencing results of genes using BLASTN from NCBI against nucleotide collection (nr / nt).**

| **Gene Name** | **Best hit in NCBI** | **Identity** |
| --- | --- | --- |
| *PPIB* | PREDICTED: Capra hircus peptidylprolyl isomerase B (cyclophilin B) (PPIB), mRNA | 100% |
| *RPLP0* | PREDICTED: Capra hircus ribosomal protein, large, P0 (RPLP0), mRNA | 100% |
| *HMBS* | Capra hircus HMBS mRNA for hydroxymethylbilane synthase, partial cds | 99% |
| *B2M* | PREDICTED: Capra hircus beta-2-microglobulin-like (LOC102168547), mRNA | 99% |
| *GAPDH* | PREDICTED: Capra hircus glyceraldehyde-3-phosphate dehydrogenase (GAPDH), mRNA | 99% |
| *18S* | Capra hircus 18S ribosomal RNA gene, partial sequence | 98% |
| *ACTB* | PREDICTED: Capra hircus actin, beta (ACTB), mRNA | 98% |
| *YWHAZ* | PREDICTED: Capra hircus tyrosine 3-monooxygenase/tryptophan 5-monooxygenase activation  protein, zeta polypeptide (YWHAZ), mRNA | 100% |
| *PPARG* | Capra hircus peroxisome proliferator activated receptor gamma2  (PPARG2) mRNA, complete cds | 100% |
