# Supplementary material for: Selection of Reference Genes for Gene Expression Studies Related to Intramuscular Fat Deposition in Capra hircus Skeletal Muscle
Source: PLoS One. 2015 Mar 20;10(3):e0121280. doi: 10.1371/journal.pone.0121280 (PMC4368700; doi:10.1371/journal.pone.0121280)
Supplement: S3 Table — (DOCX) [file pone.0121280.s005.docx]

**Table S3. Reference genes stability calculated by BestKeeper based on C_T_**

| **Longissimus dorsi muscle** | | | **Biceps femoris muscle** | | | **Combined group** | | |
| --- | --- | --- | --- | --- | --- | --- | --- | --- |
| **Gene** | **Coeff. of corr. [R]** | **Std dev [± C_T_]** | **Gene** | **Coeff. of corr. [R]** | **Std dev [± C_T_]** | **Gene** | **Coeff. of corr. [R]** | **Std dev [± C_T_]** |
| *PPIB* | 0.987 | 0.80 | *PPIB* | 0.990 | 0.76 | *PPIB* | 0.982 | 0.78 |
| *B2M* | 0.965 | 1.06 | *YWHAZ* | 0.966 | 1.34 | *HMBS* | 0.956 | 0.60 |
| *HMBS* | 0.961 | 0.60 | *HMBS* | 0.961 | 0.58 | *YWHAZ* | 0.933 | 1.42 |
| *YWHAZ* | 0.954 | 1.50 | *B2M* | 0.930 | 1.01 | *B2M* | 0.931 | 1.06 |
| *ACTIN* | 0.894 | 0.76 | *ACTIN* | 0.910 | 0.47 | *18S* | 0.829 | 1.75 |
| *18S* | 0.861 | 1.84 | *18S* | 0.876 | 1.67 | *RPLP0* | 0.780 | 0.66 |
| *RPLP0* | 0.776 | 0.68 | *RPLP0* | 0.781 | 0.65 | *ACTIN* | 0.750 | 0.84 |
| *GAPDH* | 0.704 | 0.77 | *GAPDH* | 0.615 | [0.61](http://www.wzw.tum.de/gene-quantification) | *GAPDH* | 0.661 | 1.00 |

Gene with the standard deviation (SD) value higher than 1(grayed cell) was considered inconsistent and was excluded from the calculation of the BestKeeper index. Coeff. of corr. [R]: Pearson coefficient of correlation; Std dev [± C_T_]: the standard deviation of the C_T_.
