# Supplementary material for: Selection of Reference Genes for Gene Expression Studies Related to Intramuscular Fat Deposition in Capra hircus Skeletal Muscle
Source: PLoS One. 2015 Mar 20;10(3):e0121280. doi: 10.1371/journal.pone.0121280 (PMC4368700; doi:10.1371/journal.pone.0121280)
Supplement: S4 Table — (DOCX) [file pone.0121280.s006.docx]

**Table S4. Descriptive statistics of target gene (*PPARG*) analyzed by BestKeeper in *Longissimus dorsi muscle* and *Biceps femoris muscle*, respectively.**

|  | *Longissimus dorsi muscle* | *Biceps femoris muscle* |
| --- | --- | --- |
| N | 24.00 | 24.00 |
| Geo Mean [C_T_] | 30.38 | 30.30 |
| Ar Mean [C_T_] | 30.43 | 30.33 |
| Min [C_T_] | 27.15 | 27.85 |
| Max [C_T_] | 32.99 | 32.88 |
| Std dev [± C_T_] | 1.37 | 1.12 |
| CV [% C_T_] | 4.50 | 3.71 |

Abbreviations: N: number of samples; Geo Mean [C_T_]: the geometric mean of C_T_; Ar Mean [C_T_]: the arithmetic mean of C_T_; Std dev [± C_T_]: the standard deviation of the C_T_; CV: the coefﬁcient of variance.
